# e-TEP Retromuscular Repair for Recurrent Incisional Hernias: Report of Three Cases

**DOI:** 10.1155/2019/1609193

**Published:** 2019-07-15

**Authors:** Vikal Chandra Shakya, Bikram Byanjankar, Rabin Pandit, Anang Pangeni, Anir Ram Moh Shrestha

**Affiliations:** Department of Surgery, Civil Service Hospital, Kathmandu, Nepal

## Abstract

**Introduction:**

Recurrent incisional hernias are difficult to treat. There are many factors involved in the recurrence, and due to extensive dissections, the planes are fused with adhesions, and we may need a new plane for dissection and placement of meshes.

**Case Report:**

We report here three cases of recurrent incisional hernias which were dealt by a relatively new method to laparoscopy: the enhanced view totally extraperitoneal repair (e-TEP) retromuscular technique. There were three patients: one after an open onlay repair of lower midline incision, another after an onlay mesh repair of a subcostal incision for open cholecystectomy followed by an intraperitoneal onlay mesh hernioplasty (IPOM) repair, and another after IPOM repair of epigastric hernia. They were treated with the abovementioned technique with satisfying short-term results.

**Conclusion:**

The e-TEP technique is a relatively new method of providing minimal access surgery to these patients utilizing the previously unaccessed retromuscular (Rives and/or preperitoneal) space for the repair of these recurrent incisional hernias.

## 1. Introduction

The enhanced view totally extraperitoneal (e-TEP) technique which was described mainly for laparoscopic inguinal hernia repair is now a platform for the repair of ventral and incisional hernias as well [1]. Conventional and popular surgeries for ventral hernias are open onlay mesh hernioplasty, open retromuscular mesh hernioplasty (Rives-Stoppa procedure), and laparoscopic intraperitoneal onlay mesh hernioplasty (IPOM) [2–5]. Recurrent incisional hernias are difficult to treat. There are many factors involved in the recurrence, and due to extensive previous dissections and meshes placed, the planes are fused with adhesions, and we may need a new plane for dissection and placement of meshes. Evidence seems to suggest that retromuscular mesh hernioplasty “the Rives-Stoppa repair” has advantages over other procedures regarding recurrence and functionality due to restoration of anterior abdominal wall function [3–6]. We report here three cases of recurrent incisional hernias which were dealt by a relatively new technique to laparoscopy: the e-TEP retromuscular technique, following the same principles and technical steps used by the Rives-Stoppa open technique: closure of the incisional hernia defect, approximation of the posterior layer, and mesh placement in the virgin retromuscular space [7].

## 2. Case Report

### 2.1. Case No. 1

**Figure 1:**
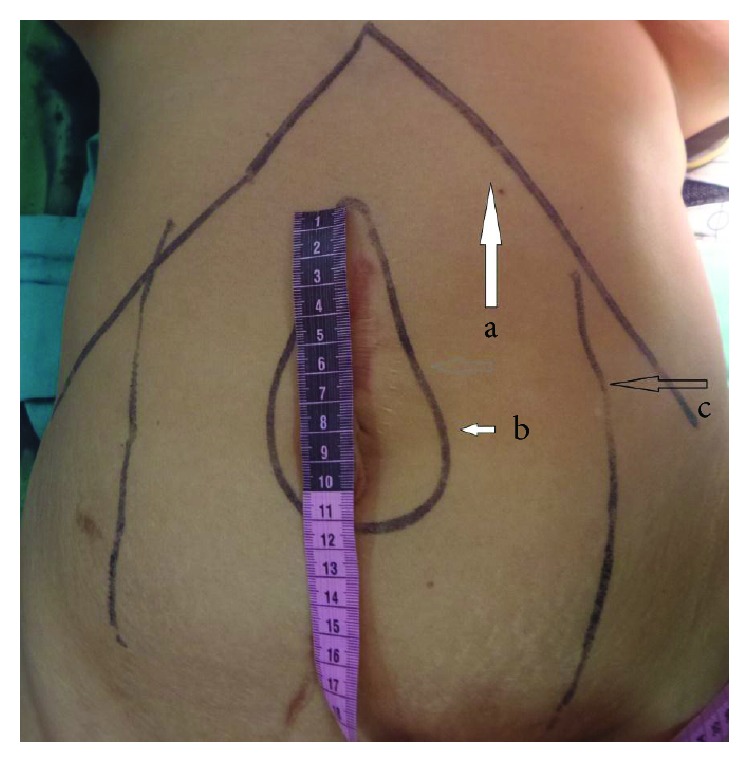
Preoperative marking of the subcostal margin (marked a), hernia defect (b), and the linea semilunaris (c).

**Figure 2:**
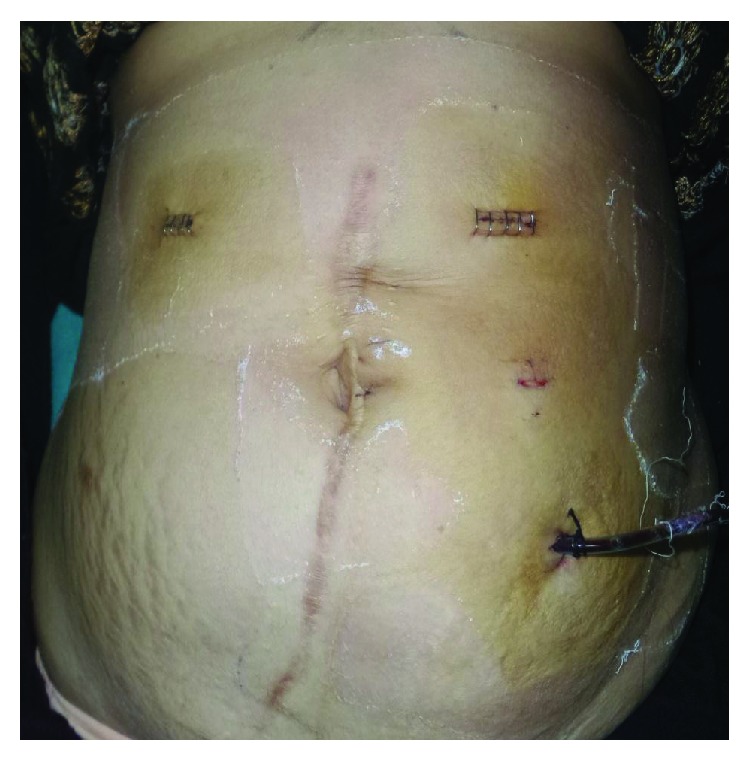
Postoperative photograph of Case no. 1 showing the port incisions.

A 39-year-old female presented with a history of abdominal hysterectomy 10 years back followed by exploratory laparotomy and adhesiolysis for adhesive intestinal obstruction 5 years back, development of incisional hernia, and repair by open onlay mesh hernioplasty 2 years back. Physical examination showed a supra- and infraumbilical incisional hernia with a periumbilical midline scar (Figure 1). She underwent ultrasonography of the abdomen; further radiological investigations could not be done. There was a defect 11 cm long and 6 cm wide around the umbilical region containing omentum. We decided to perform a laparoscopic e-TEP Rives repair in March 2018. The operative technique has been followed according to the technique put forward by Belyansky et al., except for the transfascial suture reconstruction of the linea alba [7]. The omentum was reduced, the midline defect was closed with interrupted transfascial 1-0 polypropylene sutures, and the defect in the posterior layer was closed with 2-0 polygalactin continuous without any tension. Then, a polypropylene mesh measuring 21 cm long and 14 cm wide was placed in the retrorectal space to ensure adequate overlap of the mesh edges. The previous onlay mesh did not need removal because it was in a completely different plane. She had an uneventful postoperative recovery and was discharged on the 4^th^ postoperative day (Figure 2). In 1-year follow-up, she is completely free of any complaints.

### 2.2. Case No. 2

**Figure 3:**
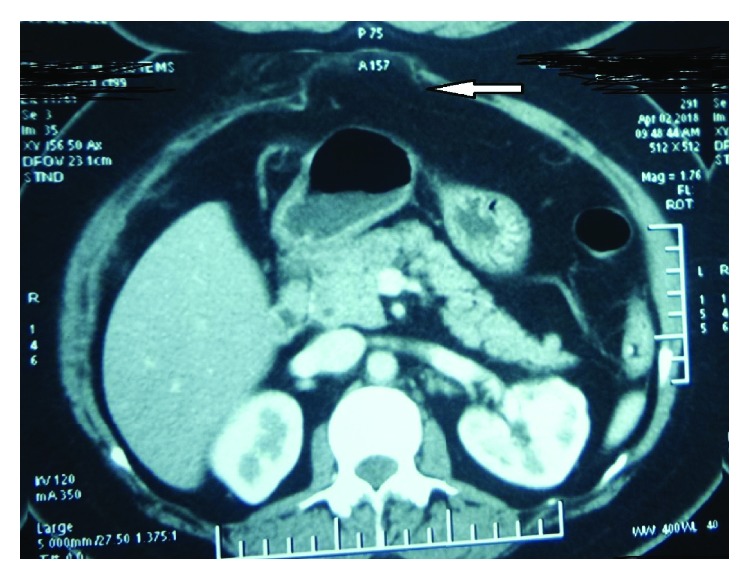
CECT of Case no. 2 showing the subcostal defect (arrow).

**Figure 4:**
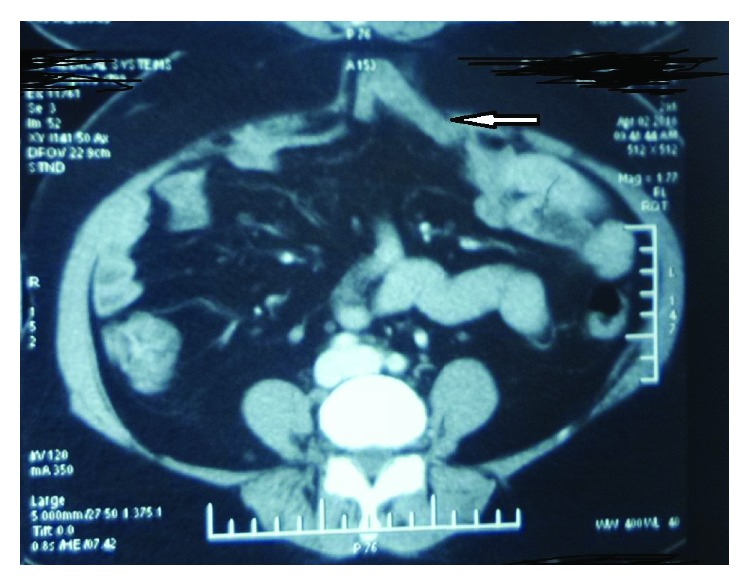
CECT of Case no. 2 showing the infraumbilical port site hernial defect (arrow).

**Figure 5:**
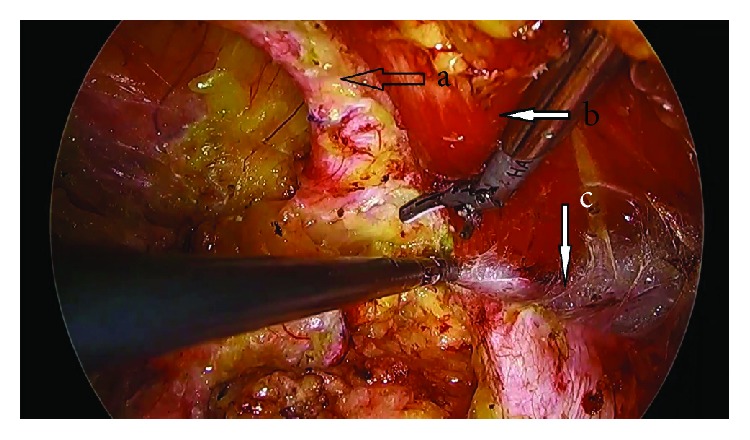
Intraoperative photograph of Case no. 2 showing the defect (marked a), the ipsilateral (left) rectus muscle (b), and the posterior rectus sheath of the ipsilateral (left) side (c) visible after retrorectus dissection.

**Figure 6:**
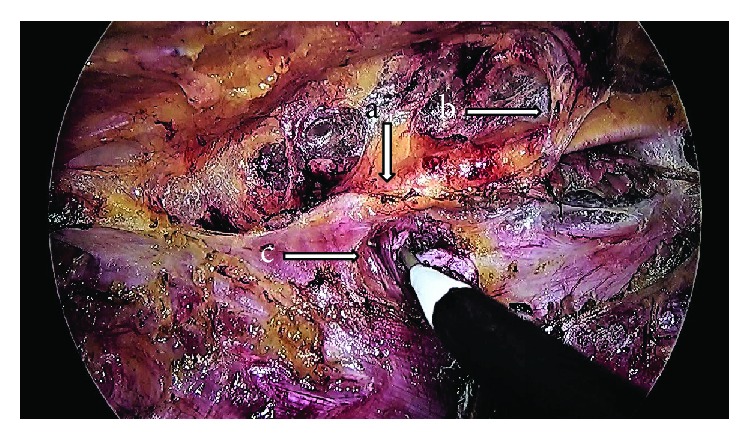
Intraoperative photograph of Case no. 2 showing the right costal margin (marked a), neurovascular bundle at the right linea semilunaris (b), and the division of the right-sided transversus abdominis muscle (c).

**Figure 7:**
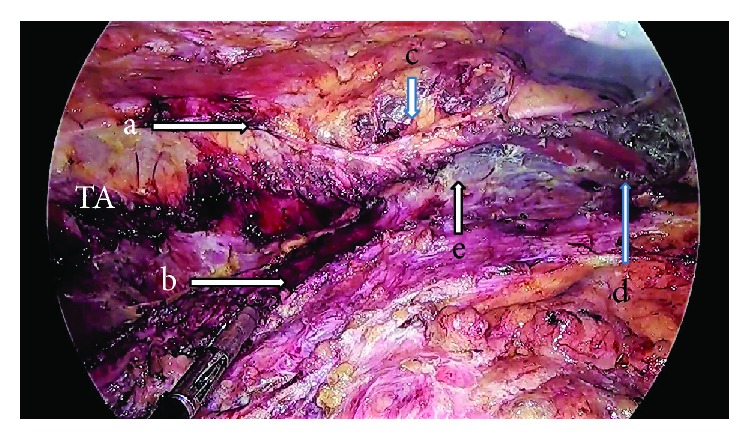
After TAR, visualization the lateral edge of divided transverse abdominis (TA) fibers (marked a), medial edge of divided TA fibers (b), costal margin (c), horizontal fibers of the diaphragm (d), the transversus abdominis muscle (labelled TA), and the retromuscular space (e).

**Figure 8:**
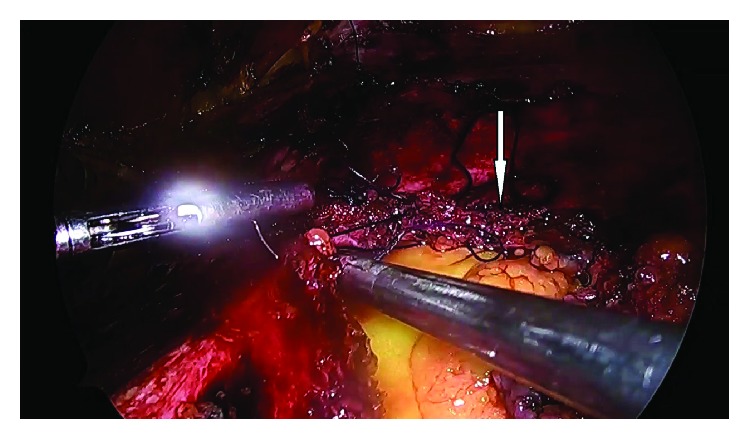
Closure of the posterior rectus sheath and the peritoneum (arrow).

**Figure 9:**
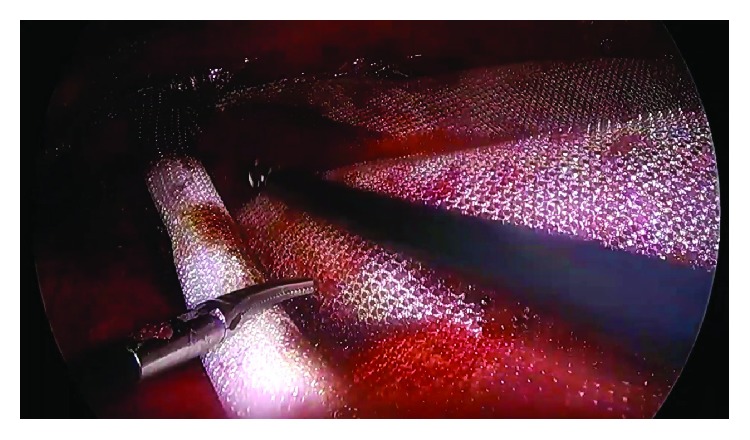
Placement and unrolling of the polypropylene mesh in the preperitoneal (retrorectus/retromuscular) space.

A 45-year-old female had a history of open cholecystectomy 19 years back and developed hernia in the medial part of the incision at the right subcostal region. She underwent an onlay mesh repair with a polypropylene mesh 8 years back which recurred after 2 years. She underwent intraperitoneal onlay (IPOM) mesh repair with a proceed composite mesh (Ethicon) tacked with a securestrap (Ethicon) and transfascial sutures 4 years back; after 6 months, it recurred again along with another infraumbilical port site hernia. She was subjected to a contrast-enhanced computed tomography of the abdomen, which showed the hernias: right subcostal hernia measuring 6 × 6 cm and the infraumbilical port-site hernia about 4 × 4 cm in size (Figures 3 and 4). In June 2018, we did a retrorectal Rives-Stoppa repair with right-sided transversus abdominis release (TAR) following the principles of Belyansky et al. [8] (Figures 5–7). After reduction of the hernia, the hernia defects were approximated with no. 1 polypropylene, the peritoneum was opposed with 2–0 polygalactin continuous (Figure 8), and retromuscular polypropylene mesh was placed (Figure 9). A component separation in the form of TAR was needed in this patient due to the unusual location of the hernia, viz. subcostal; TAR provided the space to be extended beyond the costal margin on the right side; closure of the posterior layer without tension and retromuscular/retrorectal mesh placement was possible only with the addition of TAR. Though we witnessed the previous remains of the IPOM mesh, it was found densely adhered to the omentum and intestines, so we did not try to dissect it further or to remove it. She was discharged on the 6^th^ postoperative day without any events. At 10-month follow-up, she did not have any complaints.

### 2.3. Case No. 3

**Figure 10:**
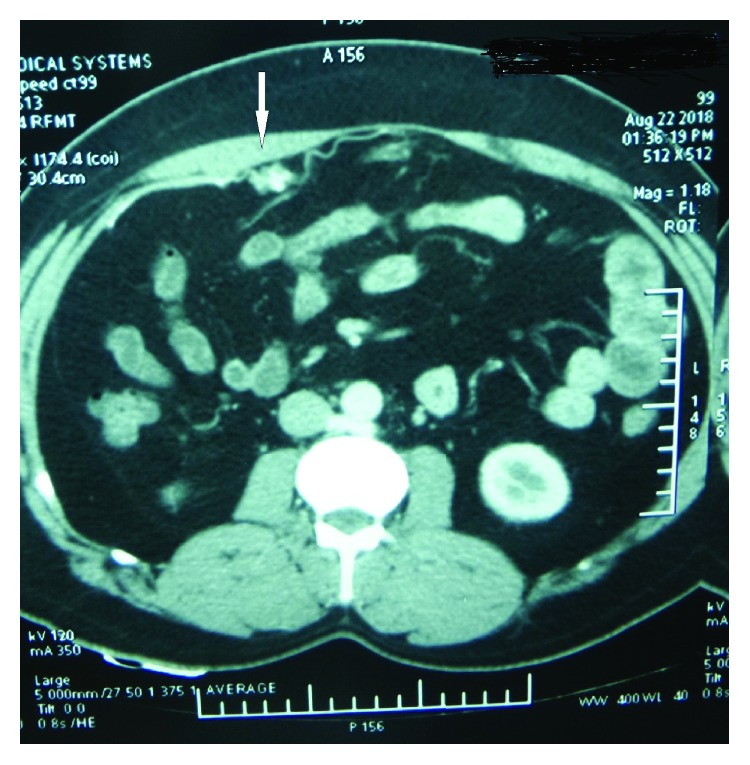
CECT of Case no. 3 showing the previously placed radiopaque displaced e-PTFE mesh placed intraperitoneally as IPOM (arrow).

**Figure 11:**
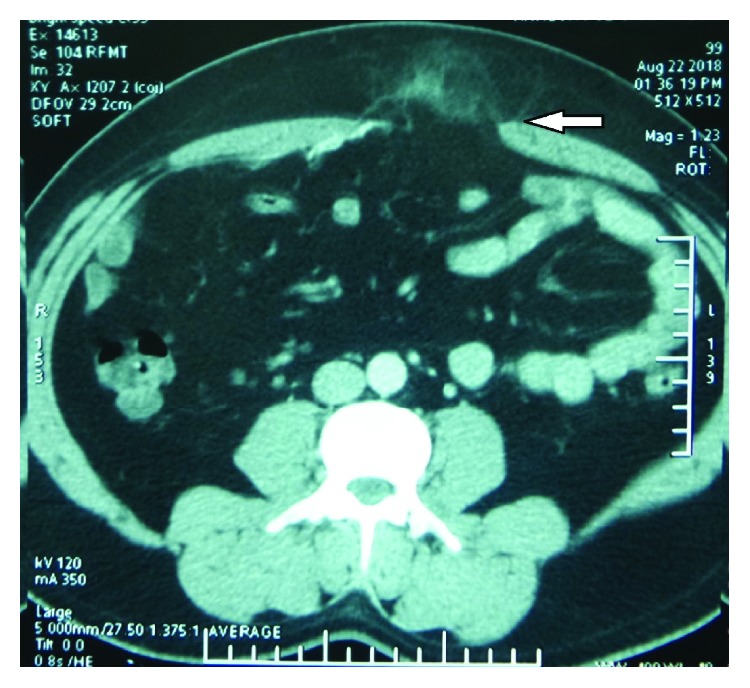
CECT of Case no. 3 showing the epigastric defect (arrow) along with part of a previous mesh.

**Figure 12:**
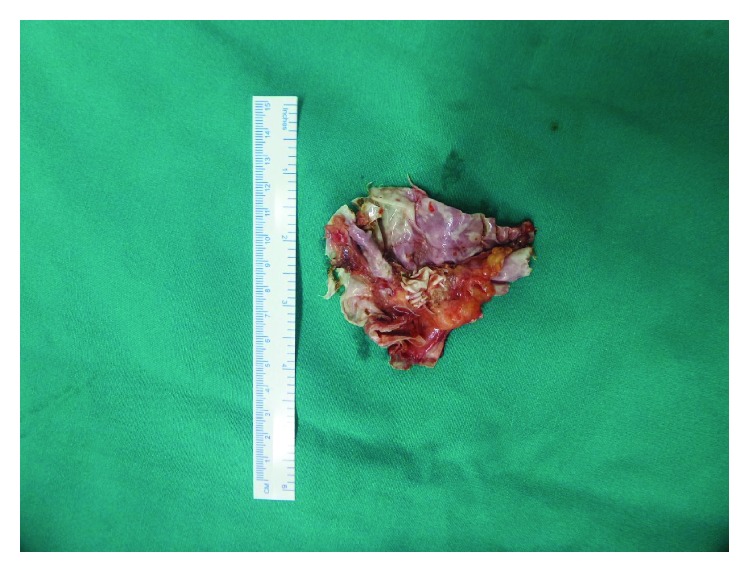
Removed e-PTFE mesh from Case no. 3.

**Figure 13:**
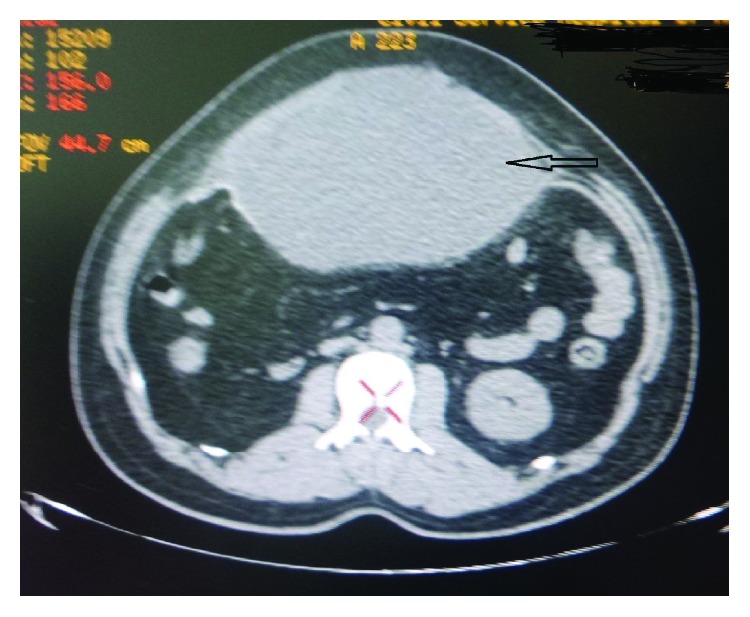
CECT of Case no. 3 showing large collection developed in the retrorectus space (arrow).

A 30-year-old male had epigastric hernia for the last 10 years; he had undergone IPOM repair with Composix™ E/X mesh (polypropylene/e-PTFE prosthesis for laparoscopic ventral hernia repair, Bard) 1 year back which recurred after 3 months. On examination, he had 6 cm × 6 cm epigastric hernia. A preoperative computed tomography scan showed the hernia with the same dimensions along with the radiopaque e-PTFE layer mesh (Figures 10 and 11). In August 2018, we did laparoscopic Rives-Stoppa repair, the removal of the previous mesh with a placement of the retrorectal polypropylene mesh (Figure 12). He developed a collection detected after 5 days, which started increasing (Figure 13); so we put a drain on the 10th day; about 600 ml of serosanguinous fluid that came out the drain was kept for 8 days more after which it was removed. The drain wound healed completely on the 20^th^ day postoperatively. Repeat CECT abdomen showed a well-apposed mesh in the parietal wall. He is completely satisfied at the 6^th^ month of follow-up.

## 3. Discussion

Since LeBlanc first reported laparoscopic approach to ventral hernia repair with the IPOM technique, its advantages were rapidly appreciated especially decreased wound complications, improved cosmesis, and faster recovery [5]. However, there are mesh- and tacker-related complications with IPOM, and the purpose of evolving laparoscopic ventral hernia repairs is avoiding the placement of a prosthetic mesh in an intraperitoneal position and the direct contact with intraperitoneal organs [6–11]. The retrorectus approach to these difficult hernias has been tested well by the open technique, and we ventured to approach if a minimally invasive technique is feasible. This takes the best advantages of a previously unaccessed new virgin plane of dissection to work upon, and with adjunct techniques like TAR, the purpose of which is to decrease the tension in the defect so that its closure is made possible; it also provides a large area for placing a mesh beyond the linea semilunaris and strengthening of the fascias with minimal transfascial fixation [8].

Meshes for hernia repair have now been accepted almost universally as a strengthening layer after inflammation and body reaction. However, there are many planes where we can put the mesh especially in ventral and incisional hernias [8–11]. The planes have been described as onlay, inlay, sublay, underlay, and intraperitoneal onlay [8–11]. In complex hernias where specific planes have been destroyed by previous surgery and mesh placements, we would have to seek a fresh plane for repair and mesh placement. The suprafascial plane in the first, the suprafascial and the intraperitoneal layer in the second, and the intraperitoneal layer in the 3^rd^ patient had all been destroyed; hence, we resorted to use the retrorectal/retromuscular plane, which was relatively virgin in these 3 patients. The anatomic location of mesh implantation does appear to influence outcomes. This extraperitoneal placement is the approach avoiding the contact of the mesh with the intraperitoneal organs hence the potential complications such as adhesions, mesh erosion, and fistulations, with an added advantage of a relatively lower cost due to the use of cheaper meshes and also lower recurrence rates in large-volume studies [8–11]. This unique feature of low-cost mesh along with a minimal access technique has encouraged us to introduce this technique of hernia repair in our developing country set-up; we have not been able to do much IPOMs as in other countries mainly due to the cost factor; this e-TEP method with the placement of a normal polypropylene mesh may see a boom in the near future in our scenario.

Difficulties are definitely present there with this relatively new technique. That is why it seems there has been a relatively long time interval between the first open Rives-Stoppa repair and the laparoscopic one, i.e., the e-TEP method [3, 4, 7]. There are particular steps in this technique which required a meticulous technique and careful dissection: (1) The first port insertion needs to be just inside the linea semilunaris; ultrasonological guidance and preoperative surface marking of the linea and margins of the defect are helpful here; we used the open technique with telescopic dissection here reaching into the retrorectal space; balloon dissection and visiport insertion have been described to assist properly as well [11]. (2) Proper dissection around the hernia defect is needed to create a working space as large as possible, because premature peritoneal puncture would lead to pneumoperitoneum and loss of the working space. (3) This requires crossover from the retrorectal space on the ipsilateral side into the opposite side space around the hernia defects and in both the supra- and infraumbilical regions; this step in the infraumbilical region is relatively easy due to the fact that ending of the arcuate line below some distance from the umbilicus ensures a continuous extraperitoneal space; however, in the supraumbilical region, it is not so easy; incision has to be made first on the medial-most part of posterior rectus sheath in the same side, dissection proceeded posterior to the linea alba and anterior to the falciform ligament, and then incision should be made on the medial-most part of the opposite posterior rectus sheath to proceed to the contralateral retrorectal space; there is a tendency to make incision much farther laterally which would lead to tension in the posterior rectus sheath when reinforcing the posterior layer; insertion of a port and a lighted telescope in the opposite space and visualization of the opposite posterior rectus sheath help this step very much. (4) The first opening of the posterior rectus sheath and entry into the peritoneum are particularly dangerous regarding the content where the potential hazard of visceral injury is present, though preoperative imaging with CECT is particularly helpful here. (5) Chances of injuring neurovascular bundles are present at the linea semilunaris, and careful dissection is needed when approaching near these structures. (6) When doing TAR, there is always a fear of making peritoneal holes, which require closure by absorbable sutures. (7) Mesh size is also particularly important; the dimensions of the final space should be measured with the help of a measuring tape; otherwise, crumbling of meshes can occur if it is large, and inadequate overlap of the defect may occur if mesh size is small. (8) Placement of a large mesh in the retrorectal space is not easy either; the operating surgeon may need to change his position from the cranial end to the caudal end and vice versa for proper mesh placement. In the 3 patients described, though the first 2 patients did not experience any complications, the 3^rd^ patient had a significant symptomatic collection in the retrorectal space; this required drain placement for few more days. Though our follow-up is relatively short (only a few months), no recurrences have been found to date; this being the main limitation of our report, a follow-up duration is very important with regard to hernia recurrence. Intraoperative complications leading to conversion to open or traditional laparoscopic techniques and postoperative complications such as recurrence, seromas needing readmissions have also been reported [7, 9, 11].

In implementing the e-TEP approach, we have followed the principles of Novitsky et al., Daes et al., and Belyansky et al. [1, 2, 6–8]. These few hernia enthusiasts have been able to produce excellent results with this novice technique. The e-TEP repair technique which we used was first described for laparoscopic inguinal hernia repair by Daes et al. and then extended to ventral hernia repairs by Belyansky et al. [1, 2, 7]. Belyansky et al. went one step further to add TAR to their series of complex abdominal wall reconstruction, both laparoscopic and robotic [8, 12]. Now, many authors believe that component separation, e.g., in the form of TAR, is needed for closure of large defects and hernia location at difficult positions such as suprapubic, subcostal, and lumbar hernias; this restores the normal anatomy, physiology, and functionality of the abdominal wall in these complex situations [13–18].

## 4. Conclusion

In patients with multiple and rerecurrent abdominal wall hernias as described in the above cases, this e-TEP approach is a feasible method with the advantages of minimal invasive approach. This provides a previously unaccessed new virgin plane of dissection to work upon and with adjunct techniques like TAR, which also decreases the tension in the anterior abdominal wall complex thereby making the closure of even large defects possible, providing a large area of a retromuscular plane for placing a mesh and strengthening the fascias with minimal transfascial fixation. Although we should wait for the long-term outcomes, this technique might be the procedure of choice for recurrent incisional hernias with minimal morbidity and low long-term recurrence rates.

## Abbreviations

e-TEP: Enhanced view totally extraperitoneal
IPOM: Intraperitoneal onlay mesh
TAR: Transverse abdominis release
TA: Transverse abdominis muscle
CECT: Contrast enhanced computed tomography
e-PTFE: Expanded polytetrafluoroethylene.
